# Dietary Vitamin B Complex: Orchestration in Human Nutrition throughout Life with Sex Differences

**DOI:** 10.3390/nu14193940

**Published:** 2022-09-22

**Authors:** Mennatallah A. Ali, Hala A. Hafez, Maher A. Kamel, Heba I. Ghamry, Mustafa Shukry, Mohamed A. Farag

**Affiliations:** 1 Department of Pharmacology & Therapeutics, Faculty of Pharmacy, Pharos University in Alexandria, Alexandria 21544, Egypt; 2 Department of Biochemistry, Medical Research Institute, Alexandria University, Alexandria 21544, Egypt; 3 Department of Home Economics, College of Home Economics, King Khalid University, P.O. Box 960, Abha 1421, Saudi Arabia; 4 Department of Physiology, Faculty of Veterinary Medicine, Kafrelsheikh University, Kafrelsheikh 33516, Egypt; 5 Pharmacognosy Department, College of Pharmacy, Cairo University, Kasr el Aini St., Cairo 11562, Egypt

**Keywords:** thiamine, riboflavin, niacin, pantothenic acid, pyridoxine, biotin, folate, cobalamin

## Abstract

The importance of B complex vitamins starts early in the human life cycle and continues across its different stages. At the same time, numerous reports have emphasized the critical role of adequate B complex intake. Most studies examined such issues concerning a specific vitamin B or life stage, with the majority reporting the effect of either excess or deficiency. Deep insight into the orchestration of the eight different B vitamins requirements is reviewed across the human life cycle, beginning from fertility and pregnancy and reaching adulthood and senility, emphasizing interactions among them and underlying action mechanisms. The effect of sex is also reviewed for each vitamin at each life stage to highlight the different daily requirements and/or outcomes. Thiamine, riboflavin, niacin, pyridoxine, and folic acid are crucial for maternal and fetal health. During infancy and childhood, B vitamins are integrated with physical and psychological development that have a pivotal impact on one’s overall health in adolescence and adulthood. A higher intake of B vitamins in the elderly is also associated with preventing some aging problems, especially those related to inflammation. All supplementation should be carefully monitored to avoid toxicity and hypervitaminosis. More research should be invested in studying each vitamin individually concerning nutritional disparities in each life stage, with extensive attention paid to cultural differences and lifestyles.

## 1. Introduction

**Figure 1:**
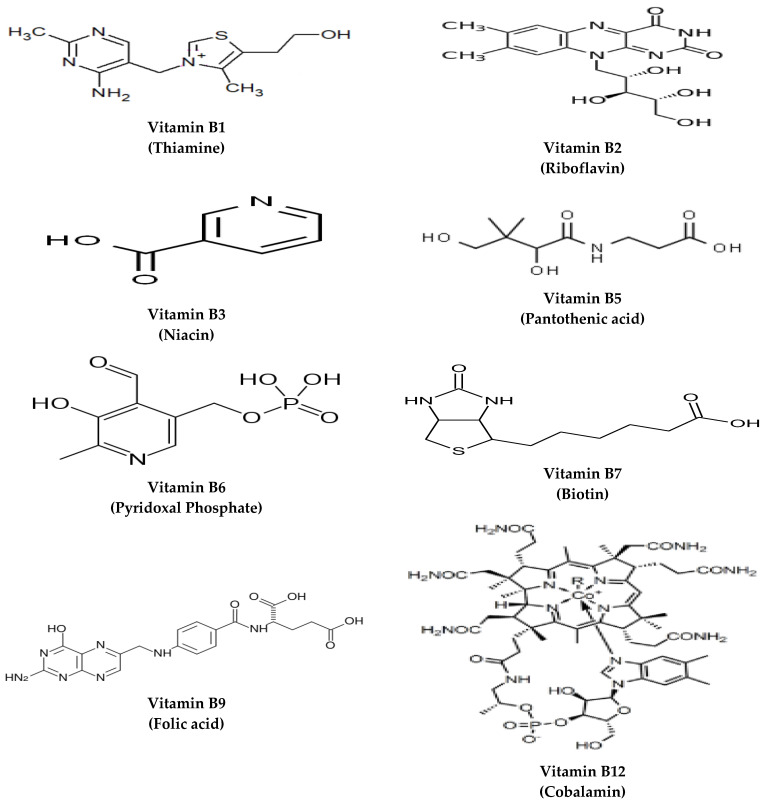
The structures of B vitamins.

Vitamins are a group of organic compounds necessary for normal physiological functions. Their established daily intake must be met as they are essential elements that cannot be endogenously synthesized. The water-soluble vitamins comprise vitamin C and B complex vitamins: Thiamine (B1), riboflavin (B2), niacin (B3), pantothenic acid (B5), pyridoxine (B6), biotin (B7), folate (B9), and cobalamin (B12). The B vitamins are not categorized according to their chemical structural resemblance, but rather concerning their water solubility and the inter-related cellular coenzyme purposes they undergo [1]. Concerning their origins, the primary source of B vitamins, except vitamin B12, is dietary food sources. Their synthesis in chloroplasts, mitochondria, and cytosol is regulated based on the plant’s changeable needs [2]. Vitamin B12 is produced by bacteria and is typically sequestered from animal-derived foods, with synthesis to occur, for example, in the foregut of ruminant animals [1]. The structures of different B vitamins are depicted in Figure 1.

Generally, it is claimed that the recommended dietary allowance (RDA) of vitamins during the life cycle may differ owing to the variation in both metabolic and functional demand for vitamins at this stage. Hence, humans in different locations might be vulnerable to the risk of inadequacy, and its impact on human health varies according to the extent of deficiency [3].

For instance, vitamin B12 deficiency can be manifested as megaloblastic anemia that might progress to funicular myelosis as an end-stage disease [4]. The risk of developing such deficiency exponentially increases in stages of higher demand, such as pregnancy and early childhood. In addition, pregnancy is a critical stage in life, requiring adequate micronutrient supplementation.

Special consideration is given to folate to prevent the risk of neural tube defects [5]. Moreover, the risk of having a small-for-gestational-age child is fourfold in pregnant women with inadequate diets, especially in folate and ferritin.

Herein, the current review presents the most updated information from the scientific literature regarding B complex vitamins’ dietary requirements, metabolism, interaction, and importance in different life stages. At first, each dietary vitamin source, metabolism, and associated deficiencies are presented. Next, analysis from recent research on the diverse needs of vitamin B complex is offered at different life stages, i.e., preconception, pregnancy and lactation, pediatrics, adult, and old life, and in the context of sex type, i.e., male and female.

## 2. B Vitamins Orchestration in Different Life Stages

As mentioned above, during human life, the daily requirements of B vitamins vary according to the demands of each life stage.

During the critical stage of pregnancy and lactation, maternal balanced nutrition and adequate micronutrient intake have a crucial impact on the health and survival of both the mother and fetus before and during the gestational period and later through their life. This stage is characterized by profound and rapid physiological alterations that might predispose women to disorders such as obesity, insulin resistance, and calcium deficiency associated with osteopenia [6]. In addition, physical and cognitive functions during childhood and leading up to adulthood can be affected significantly due to poor maternal nutrition and inadequate intake of micronutrients such as B vitamins [7]. Insufficient nutrition and micronutrient deficiency contribute to depression and stress, affecting the mother’s health and, consequently, her baby’s status [8], which in turn obliges a nutrient-dense diet and social support. It is noteworthy that although some vitamins are crucial during these stages of life, others, such as biotin significance, were not reported during pregnancy and lactation, which is likely attributed to its endogenous synthesis, which makes biotin deficiency and complications very scarce at such life stages.

In infancy, evidence has shown that environmental factors such as diet and physical activity can affect infants’ physiological and psychological development, affecting their health and longevity in adulthood. There is also a growing interest in comparing breast and formula feeding in early infancy and their impact later in life, particularly concerning developing diseases such as obesity, hypertension, hyperlipidemia, and diabetes mellitus [9].

Later in childhood, developing good habits for living a healthy lifestyle can help improve their performance in school and other adult life stages, in addition to preventing later-life non-communicable diseases such as obesity and all related metabolic disorders, pulmonary disorders, anemia, and dental caries [5]. Addressing nutritional disparities among pediatric populations is an international endeavor. However, it can help guide future research and development of policies and programs aimed at fulfilling the daily requirements of vitamins and minerals during this stage of life, especially B vitamins.

In adulthood, good nutrition also plays a crucial role in attaining and sustaining a healthy lifestyle due to the complex link between diet and health. Nutrition at this stage is often linked to various factors such as biological, environmental, and cultural factors and is often a reflection of the early nutritional status [10]. The potential risks of weight loss, cardio-respiratory disorders, gastrointestinal-related disorders, impaired immune response, and wound healing increase with malnutrition [11].

In the elderly, it is worth mentioning that during the next five decades, the proportion of people over 60 is expected to rise from 11% to 22%, estimated to be 2 billion [12]. Hence, it is imperative nowadays to focus on the nutritional status of the elderly population to prevent the risk of Alzheimer’s disease, diabetes, obesity, insulin resistance, metabolic syndrome, and cardiovascular diseases [13], with careful attention to the recommended daily intake of B vitamins. In addition, other disorders are also related to malnutrition in the elderly such as osteoporosis, skeletal muscle wasting, and impaired wound healing. All these are related to inflammation and immunosenescence due to aging [14].

**Figure 2:**
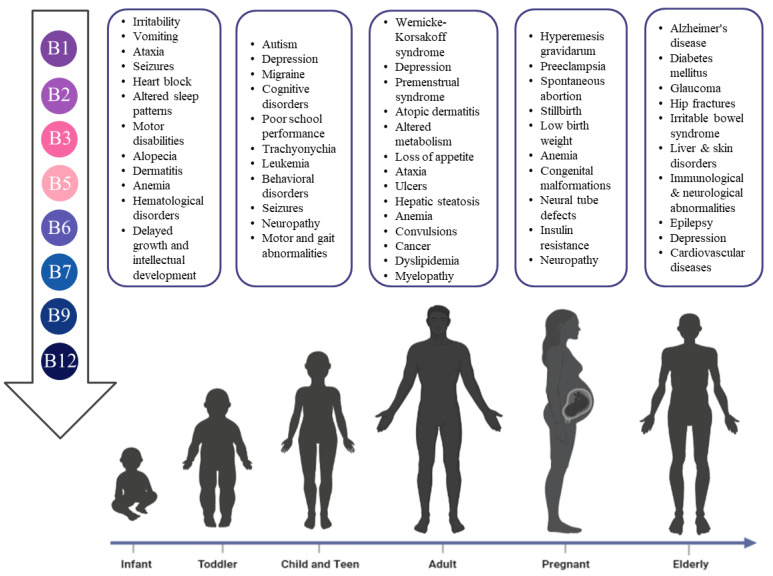
The risks of B vitamins deficiency in different life stages.

Though a sufficient quantity of the B vitamins should be attained from a healthy diet, growing evidence suggests that humans at specific locations have increasing demands of one or more of the B vitamins, such as childhood or pregnancy, which might pose a negative impact on overall health and or brain functions as explained in detail in the following subsections. The deficiency of these vitamins in different life stages is crucial and might predispose humans to a multitude of disorders, as illustrated in Figure 2.

### 2.1. Thiamine

The recommended daily intake, main action, and natural sources of vitamin B1 in different life stages are illustrated in Table 1.

Pregnancy and lactation: Thiamine deficiency in pregnancy can result from an extreme case of nausea and vomiting known as *Hyperemesis gravidarum* (HG), which could lead to Wernicke’s encephalopathy (WE) and Korsakoff syndrome (KS). The most observed WE symptoms are eye movement disorders, which were present in 86.4% of cases, altered mental status or cognitive abilities in 83.6%, and ataxia in 83.1%. Treatment of HG comprises 700 mg/day parenterally of thiamine and lower doses (435 mg/day) of thiamine for HG cases with altered cognitive abilities [15].

Thiamine deficiency most commonly occurs during lactation, pregnancy, and increased physical activity. The central nervous system oversees 20% of the body’s metabolic activity and therefore has high-energy requirements, with brain energy primarily coming from glucose. Impaired glucose metabolism is thus known to cause neurological illnesses, necessitating thiamine supplementation for both mother and infant health [16].

Infancy: Infantile thiamine intake for the first six months of life depends on maternal intake. Maternal supplements are recommended to avoid impaired infant growth in the case of deficiency. Thiamine in human milk exists as either free thiamine or thiamine monophosphate, with the phosphorylated forms unable to pass through membranes [16]. Exclusively breastfed infants have the risk of thiamine deficiency. The risk of thiamine deficiency increases with delayed complementary feeding. Infantile thiamine deficiency could also occur due to the consumption of soy-based formula, especially in the case of vegetarian-based diets that are thiamine-deficient [17]. In infancy, thiamine deficiency could cause irritability, vomiting, ataxia, and altered sleep patterns. In addition, long-term consequences of thiamine deficiency in infants are motor disabilities, seizures, heart block, and delayed motor skills [18].

Regional studies for women in Kashmir that relied on polished rice and meat or chicken soups were found to lead to maternal thiamine deficiency, especially in times of stress such as pregnancy and lactation, in which levels are already depleted. A study conducted in 2014–2015 in Kashmir reported infantile encephalopathy, which was related to thiamine deficiency. The study included 50 exclusively breastfed 1–6 months infants with acute encephalopathy, with 90% of the subjects of the lower socioeconomic class [19]. Infant encephalopathy could develop rickets, viral and bacterial infections, urea cycle defects, and fatty acid oxidation disorders.

Childhood: A previous study linked thiamine deficiency with autistic features in children. In contrast, a thiamine derivative (thiamine tetrahydrofurfuryl disulfide) administered 50 mg twice daily for two months improved social skills and cognitive awareness [20]. Moreover, depression in children was witnessed, as mood swings or laziness were also suggested to be linked to thiamine deficiency [17].

Adulthood: Thiamine deficiency is often associated with alcohol abuse. Alcohol metabolism leads to reduced transport of thiamine to areas in the body, including the blood–brain barrier. The enzymes involved in brain cell metabolism, such as α-ketoglutarate and pyruvate dehydrogenase, which are dependent on thiamine coenzymes, are present at lower levels in alcoholic patients with Wernicke–Korsakoff syndrome. In addition, alcohol-induced liver damage also reduces the storage and phosphorylation of thiamine, thus contributing to its deficiency [21]. Considering that this life stage is at which alcohol consumption starts, the measurement of thiamine levels ought to be considered.

In Wernicke–Korsakoff syndrome in alcoholic patients, thiamine supplements are typically administered as a dose of 500–1200 mg per day intravenously or via intramuscular injection over 2–3 doses for five days, then an oral dose of 300 mg per day for 1–2 weeks, and finally 100 mg for maintenance. Hence, it is recommended that alcoholic patients should be supplemented with thiamine at a dose of 100–300 mg per day [22].

Abruptly stopping alcohol intake with thiamine deficiency could increase glutamate function and release, which could cause neurotoxicity due to glutamate hyperactivity. Thus, it is suggested that alcohol withdrawal should be accompanied by thiamine supplementation to detoxify the brain [21]. Adult depression, which might be a consequence of alcohol consumption, was also reported to reduce thiamine bioavailability [17].

An exciting application of thiamine is lessening the symptoms of premenstrual syndrome (PMS), which occurs in 85–90% of women of reproductive age. The symptoms can be behavioral (mood swings, depression, anxiety, and tension) or physical (abdominal cramps, fatigue, flushing, and dizziness) with different degrees of severity. PMS is hypothesized to be caused by an alteration in dopamine and serotonin levels, aldosterone activity, and essential fatty acids. Thiamine is also used to treat nausea, vomiting, muscle cramps, and anxiety, which are all symptoms similar to PMS. Thiamine is involved in carbohydrates and amino acids metabolism, as well as neurotransmitters biosynthesis that could, in part, lessen PMS symptoms.

In the same context, thiamine was investigated in menorrhagia, which occurs when bleeding lasts for more than seven days or if the blood volume is over 80 mL and affects mental and social health and might lead to anemia. A double-blind clinical trial was conducted in Iran in 2016–2017 on female students aged 18–26. The study showed that thiamine could treat menorrhagia with minimal side effects, manifested as a reduction in the duration of menstrual bleeding. While the mechanism is not fully known, thiamine represents a safe and inexpensive way to manage menstruation symptoms and improve female adults’ productivity [23].

Elderly: Furthermore, impaired cerebral glucose metabolism indicates Alzheimer’s disease (AD) is likely to occur in the elderly stages. Thiamine deficiency causes beriberi, WE, and Korsakoff Syndrome, exhibiting symptoms of ataxia, mental confusion, and later amnesia, similar to AD. AD patients exhibit reduced α-ketoglutarate and pyruvate dehydrogenase enzyme activity and levels linked to thiamine deficiency. It was also concluded that lower TDP levels could be a predisposing factor for AD that is also gender-related [24]. In contrast, another study failed to show any thiamine deficiency in a group of elderly patients. However, this might not be indicative as low thiamine metabolism was not investigated, nor was there any evidence of supplement consumption among subjects [25].

### 2.2. Riboflavin

The recommended daily intake, its primary action, and natural sources of vitamin B2 are presented in Table 2.

Pregnancy: Riboflavin deficiency appeared as a possible risk factor for preeclampsia. Insufficient levels of riboflavin-derived cofactors Flavin mononucleotide (FMN) and Flavin adenine dinucleotide (FAD) could contribute to the established pathophysiologic changes in preeclampsia, including mitochondrial dysfunction, enhanced oxidative stress, and disturbances in nitric oxide release [26]. In lactation, the average intake of mothers is 0.39 mg/L, with 0.01–0.55 mg secreted in breast milk. Such intake is lower than the RDA, which is 1.6 mg/L. Lactating mothers should thus increase riboflavin consumption to avoid its depletion. Unlike thiamine, riboflavin levels in breast milk remain consistent over time [27].

Infancy: Indications have been reported for riboflavin being important in the early postnatal development of the brain. Riboflavin can be destroyed by UV light exposure; thus, UV therapy in infants with hyperbilirubinemia could result in riboflavin deficiency. Infant nutrition could be improved by adding weaning food fortified with riboflavin to reach up to 0.4 mg/day [16].

Childhood and adolescence: Complementary and alternative medicine is recently being used to treat children and adolescent diseases, i.e., migraine, pain, and attention deficit hyperactivity disorder (ADHD). Alternative medicine to treat migraines includes riboflavin, magnesium, coenzyme Q10, and others. Since riboflavin plays a role in mitochondrial energy production as a coenzyme in the flavoprotein redox reactions, it could reduce migraine attacks regarding mitochondrial malfunction. Nevertheless, clinical trials failed to demonstrate a significant difference in migraine severity or frequency when comparing riboflavin and placebo. An observational test administered at a dose of 20–400 mg riboflavin per day to children and teenagers (7–18 years) for six months who suffered from migraines showed a reduction in the frequency and intensity after 3–4 months. Still, it showed a non-significant difference between the placebo and riboflavin groups [28].

Adulthood: Riboflavin should also be considered in adult migraines, similar to childhood, which is similarly associated with mitochondrial dysfunction. Only 27 mg of the riboflavin, at most, is absorbed per dose with a ca. one h half-life. An administration of 50–400 mg/day of riboflavin concluded that only some adult populations would be able to benefit from the treatment due to genetic variability [29]. Similar to thiamine, riboflavin intake in women was also associated with a decreased incidence of PMS [30].

The malfunction of one-carbon metabolism, which involves reactions donating methyl groups, is one factor that can promote breast cancer’s carcinogenesis. Riboflavin is a cofactor for some enzymes involved in these reactions, so it might play a role in reducing the risk of breast cancer. However, a meta-analysis that analyzed ten studies from 1966 to 2016 with a total of 12,268 cases aged between 20 and 80 years failed to demonstrate the ability of riboflavin to decrease breast cancer risk. The analysis was limited by the number of detailed studies [31].

Elderly: Riboflavin could also reduce the risk of Type 2 diabetes (T2DM) in the elderly as a common degenerative disease. T2DM is associated with oxidative reactions that lead to insulin resistance. The exact mechanism of riboflavin is not fully elucidated, but its preventive role could be attributed to its antioxidant activity and the ability to reduce iron overload. A study examining 19,168 healthy Japanese men and women for five years concluded that a 44% reduced risk of T2DM was associated with riboflavin intake of 1.8 mg/day, but only in women [32].

### 2.3. Niacin

Vitamin B3 has diverse functions and dietary sources, as depicted in Table 3.

Pregnancy: Pregnant women are more susceptible to micronutrient deficiency; their RDA is higher than ordinary adults, increasing from 14 mg niacin equivalents (NE) to 18 mg NE, ca. 25–30% extra dose intake. By studying the whole human exome, variation was found in the de novo kynurenine pathway (HAAO gene, encoding 3-hydroxyanthranilic acid 3,4-dioxygenase, and KYNU gene, encoding kynureni-nase). An evident relationship between niacin deficiency and multiple congenital malformations in the fetus was observed. Both participate in the NAD synthesis, resulting in NAD deficiency leading to VACTERL malformations (vertebral anomalies, anorectal malformations, cardiovascular anomalies, tracheoesophageal fistula, esophageal atresia, renal and/or radial anomalies, and limb defects). Preclinical studies on rats showed that mutations disappeared in embryos upon niacin supplementation with mothers [33,34], which shed light on the value of gestational niacin intake and the necessity to measure NAD levels in the body [35].

Lactation: Exclusive breastfeeding is recommended for the first six months after birth. Therefore, any maternal micronutrient fluctuations may affect growth and development at future stages. Unlike minerals, maternal intake and stores affect vitamin levels in breastmilk. Any deficiency in the mother’s niacin levels consequently affects nicotinamide levels in breastmilk [36], which is the primary form of niacin in breastmilk.

Infancy: Niacin is essential for normal infancy development and growth. Its content in human milk is approximately 1.5 mg/L, whereas its precursor tryptophan content reaches 210 mg/L. Hence, the total content is ca. 5 mg niacin equivalents/L secreted daily in human milk [37]. Similar to niacin, the adequate intake of pantothenate is 1.7 mg/day in infants, which is crucial for average growth and intellectual functions [38].

Adulthood: Adequate niacin levels can also prevent the occurrence of pellagra-associated dermatitis. Unexpectedly, supplemental niacin intake did not necessarily decrease the risk or prevent atopic dermatitis in women to the extent that increased additional niacin intake was positively associated with atopic dermatitis in some cases [39]. However, topical or orally administered nicotinamide was associated with restoring epidermal barrier integrity, which is usually impaired in atopic dermatitis patients [40]. Further studies addressing this topic in different geographical areas and medical histories are eagerly required.

Increased niacin intake with vitamin B6 and folate in young adulthood positively affected cognitive functions in later life. Cognitive functions were improved with niacin intake of more than 17.53 mg/1000 kcal. Such significance diminished with higher education groups of people, suggesting that race, gender, and educational level are contributing factors [41]. Another study comprising 127 adults of men and women showed that higher niacin intake was positively related to arterial flow-mediated dilation that might result in reduced LDL levels and associated oxidative stress [42].

In addition, it is recommended that niacin RDA for athletes aged 19 to 25 years is 22.8 mg for women and 30.36 mg for men with the maintenance of their recommended caloric intake [43].

### 2.4. Pantothenic Acid

Despite its importance, the abundance of pantothenic acid dietary sources and the rarity of its deficiency cases have led to limited research on its importance in the different life cycle stages. Its significance and nutritional sources are listed in Table 4.

Pregnancy: Pregnant females can maintain the average blood level via increased calorie intake or more carefully selecting foods rich in pantothenic acid. On the other hand, increased pantothenic acid intake of more than 5.6 mg/day and high biotin and riboflavin intake above 22.5 μg/day and 2.42 mg/day, respectively, are associated with genome instability, profoundly manifested during pregnancy, and could lead to teratogenicity [44].

Childhood and adulthood: Although vitamin B5 deficiency is rare, a lack of this vitamin can adversely affect a wide range of metabolic functions, such as energy production and reduction in the synthesis of lipids. Symptoms vary from a loss of appetite, growth impairment, dermatitis, weakness, and ataxia to paralysis, adrenal hypertrophy, ulcers, and hepatic steatosis, which are usually misdiagnosed as a deficiency in other vitamins, delaying proper treatment. [45].

Elderly: Higher levels of pantothenic acid were also suggested to be associated with increasing cerebral amyloid-β peptide burden in cognitive impairment patients, significantly worsening cases of Alzheimer’s disease [46]. Still, more comprehensive scientific research needs to be directed towards such controversy by analyzing larger subject groups over extended periods to clarify the mechanism underlying such adverse effects.

In contrast, by examining 908 subjects aged more than 40 years over five years, pantothenic acid was found to be inversely related to the levels of the C-reactive protein, which is an indicator of low-grade inflammation and plays a critical role in the development and progression of atherosclerosis [47].

### 2.5. Pyridoxine

Table 5 illustrates the importance of vitamin B6, its RDA in different stages, and its dietary sources.

During pregnancy, the placenta produces alkaline phosphatase (ALP), which hydrolyzes the active form of vitamin B6 (PLP) into pyridoxal resulting in decreased levels of vitamin B6 [48]. Consequently, a higher intake of vitamin B6 in pregnant women (population reference intake of 1.8 mg/kg) is recommended to maintain adequate serum PLP levels [49]. It was also suggested that daily administration of 5.5–7.6 mg of pyridoxine would be sufficient to optimize pyridoxine levels of a pregnant female [50]. Non-pregnant women taking oral contraceptive agents (OCAs) share the same high demands for vitamin B. During the OCAs course, estrogen stimulates the aminotransferase enzyme, which causes abnormalities in vitamin B6 metabolism leading to its deficiency [51,52].

The significance of Vitamin B6 for a pregnant female is extended from pregnancy stabilization during the first trimester to mood elevation after birth, including nausea and anemia improvement. Monitoring pyridoxine levels during the first trimester is essential to decrease nausea and vomiting, maintain pregnancy, and prevent miscarriages. Typically, pregnancy is stabilized by the action of a placental enzyme called diamine oxidase, whose activity is controlled by vitamin B6. Consequently, pregnant women with pyridoxine deficiency may experience spontaneous abortion and stillbirth [53], which warrants a vitamin B6 supplement prescription for females with frequent miscarriages. Moreover, the low level of methylene tetrahydrofolate reductase enzyme that converts homocysteine (Hcy) into methionine was reported in these females, leading to high levels of Hcy, which results in spontaneous abortion [54]. Secondly, a deficiency in vitamin B6 has been linked to hyperemesis gravidarum, nausea, and vomiting [55]. Vitamin B6 supplements in doses of up to 510 mg/day effectively improve such symptoms without increasing the risk of fetal malformation [56,57]. More studies are still needed to unveil the molecular and biochemical mechanism of how vitamin B6 improves these symptoms.

One of the most frequent complications among pregnant women is iron-deficiency anemia. However, some anemic pregnant women are non-responsive to iron supplementation. Surprisingly, anemia in those patients was shifted after vitamin B6 administration. Vitamin B6 is not only essential for heme and porphyrin synthesis but also for proper iron utilization by red blood cells [48]. Thus, vitamin B6 inadequacy can be linked to anemia in general and iron-deficiency anemia specifically. Therefore, an appropriate diagnosis of anemic pregnant women requires monitoring vitamin B6 and iron levels. Pyridoxine supplementation in a dose of 80 mg/day before birth at gestational week 28 and 40 mg/day after birth can be used as mood elevators to overcome postpartum depression [58].

The maternal intake of vitamin B6 determines the fetal status of pyridoxine. Therefore, it has been recommended to administer more than 4 mg/day of pyridoxine during pregnancy to maintain adequate PLP levels that ensure normal fetal development [59].

Lactation: Vitamin B6 also plays a crucial role in average infant growth in height and weight [60]. Therefore, pyridoxine RDA for lactating mothers is the same as for pregnant females. Moreover, mothers with deficient vitamin B6 levels are advised to provide their newly born with vitamin B6 supplements [61]. Puerperal lactation and hyperprolactinemia are some problems lactating mothers face due to excessive prolactin release [62]. Since L-dopa stimulates prolactin release, it is predicted that vitamin B6 may be effective in prolactin reduction by converting L-dopa to dopamine [62]. However, more research is needed to confirm this hypothesis.

Infancy: The glutamic acid decarboxylase enzyme maintains the balance between the glutamate and the inhibitory GABA excitatory neurotransmitter glutamate. Some infants are born with an autosomal recessive defect in this enzyme, which will shift the balance toward excitatory glutamate. This state of excitation is seen as non-responsive polymorphic seizures. Pyridoxine administration at a dose of 10–300 mg/day showed significant improvements, whereas seizure recurrence was observed after pyridoxine termination. Hence, lifelong treatment with vitamin B6 supplements is essential for those infants [63].

Childhood and adolescence: Pyridoxine is required for thymidine biosynthesis and host immunocompetence; therefore, it has a role in carcinogenesis and tumor growth [64]. Vitamin B6 regulates the synthesis and metabolism of 5-hydroxy tryptamine (5HT), serotonin receptors, and catecholamines. Thus, it is widely used to treat the symptoms of behavioral disorders during childhood. For instance, it improves abnormal behavioral conditions associated with autism, hyperkinetic syndrome, and schizophrenia [65,66]. Moreover, it is an effective adjuvant to anti-epileptic drugs, such as levetiracetam, to prevent agitation, irritability, and depression [67].

Furthermore, vitamin B6 was reported to exert beneficial effects on stress accompanying the adolescence phase owing to its role in facilitating magnesium (Mg) uptake. Stress is associated with low Mg levels; however, providing magnesium alone was ineffective in achieving calmness and relaxation. As vitamin B6 facilitates Mg uptake, pyridoxine is given in adjunct with magnesium at a ratio of 10 Mg: 1 Vitamin B6. Moreover, pyridoxine reduces corticosteroid release peripherally and affects the central biosynthesis of various neurotransmitters related to depression and anxiety, meaning it is suggested as an anti-stress agent at a dose of 100–300 mg/day [68].

Adulthood: Vitamin-B6-deficient adults may develop microcytic hypochromic anemia, termed pyridoxine-responsive anemia and remediable by pyridoxine only [69]. Lymphopenia is a disease where T-cells percentage is significantly reduced, leading to poor immune responses. In adults, vitamin B6 deficiency affects immune response negatively by decreasing T-helper levels. Pyridoxine intake at a dose more significant than the recommended for this age group, reaching 50 mg/day, is adequate and highly recommended in such cases [70].

Similar to in infants, vitamin B6 insufficiency decreased GABA levels and increased nerve excitability. During adulthood, convulsions caused by vitamin B6 deficiency are either due to poor intake, liver disease, pregnancy, or certain medications, with seizures found to be immediately remedied by adequate pyridoxine intake. Adults with tuberculosis and under isonicotinic acid hydrazine (INH) treatment may develop pyridoxine-dependent seizures attributed to the effect of INH metabolite in inhibiting the pyridoxine phosphokinase enzyme, leading to reduced PLP levels [71].

In contrast to pyridoxine’s effect on childhood leukemia, it is incredibly beneficial against colorectal cancer in adult males. This is justified by the action of PLP in inhibiting RNA polymerase, RNA reverse transcriptase, and DNA polymerase, which are overly expressed during increased cellular proliferation and oncogenic transformation [72].

The carnitine palmitoyl transferase enzyme is essential in controlling the availability of long-chain fatty acids for mitochondrial oxidation. It is synthesized endogenously in the liver and kidneys in the presence of vitamin B6. Consequently, carnitine biosynthesis is decreased with vitamin B6 deficiency concurrent with fatty acid accumulation and lipid profile alteration. Therefore, vitamin B6 is given to men with hypertriglyceridemia to reduce plasma cholesterol levels [73].

For both males and females, marginal vitamin B6 restriction decreases the plasma concentration of long-chain PUFA, n-3, and n-6, and increases the chance of developing CVD [74].

Elderly: Poor nutritional status was associated with low B6 levels, albeit B6 deficiency also occurred in subjects without malnutrition. Old age, inactivity, low serum albumin, alanine aminotransferase levels, and high homocysteine levels were associated with pyridoxine deficiency. Irritable bowel syndrome was also correlated with vitamin B6 deficiency [75].

### 2.6. Biotin

Biotin has necessary human functions, which are shown along with its RDA and dietary sources in Table 6.

Infancy: During infancy, vitamin B7 is critical in maintaining healthy hair, skin, and nails and preventing severe brain abnormalities. Biotin insufficiency in infancy is linked to alopecia and dermatitis around body orifices. Symptoms are reversed with a daily dose of 1 mg biotin. Since no recurrence was observed when biotin was stopped gradually, patients can gradually decrease the dose to 0.5 mg, 0.25 mg, and 0.1 mg over seven months [76,77,78,79]. Biotin is advised not to be abruptly stopped as it might cause sudden infant death syndrome (SIDS). The relationship is based on clinical observations that hepatic biotin level in infants who died from SIDS was lower than in infants of similar age who died of explicable causes [80,81]. However, these clinical studies are still insufficient, and further investigations are indispensable to reveal the exact causes of SIDS and unveil the precise attribution of biotin deficiency in these cases.

Childhood: Biotin supplementation at a dose of 2.5 mg/day for 180 days was efficient in both shiny and opaque types of trachyonychia. Trachyonychia, also called dystrophy, is an abnormal nail plate roughness that occurs during childhood and has been linked to biotin deficiency [82].

Adulthood: Biotinidase deficiency is one of the causes underlying biotin deficiency in adults. Since it is an inherited disorder, neonates should be screened early to avoid its delayed onset in adulthood, manifesting as myelopathy and irreversible neurological damage [83].

Elderly: Additionally, biotin deficiency is connected to specific diseases such as diabetes mellitus, liver and skin disorders, immunological and neurological abnormalities, and epilepsy. Moreover, it plays a role in bone mineral homeostasis, which is critical in such elderly life stages, and allergic and autoimmune disorders via biotinyl IgG [84].

### 2.7. Folic Acid

Vitamin B9 has indispensable significance in human health in different life stages, which is presented in Table 7.

Pregnancy and lactation: Folic acid is a well-known supplement in pregnancy because of its prominent role in the fetus’ normal neural and physical development. Folate deficiency in the gestational period resulted in severe fetal adverse effects, including congenital neural tube defects, cardiac and urinary tract defects, and even cancer [85], besides affecting birth weight [86]. The development of neural tube defects from altered levels of neurotransmitters and limited myelination leads to the long-term impairment of cognitive function, learning, and memory deficits and brain atrophy detected in infants [86]. Its deficiency also might cause metabolic effects such as insulin resistance, glomerular sclerosis, neuropathy in the extremities, and megaloblastic anemia in the mothers [87].

The folate daily requirement during pregnancy and lactation is higher than normal, reaching 600–800 µg daily to meet the higher demands of the growing fetus and infant. During lactation, women risk folate deficiency due to increased demands to accommodate milk folate levels [88]. Moreover, maternal folate levels are critical factors for the proper development of the offspring.

The hyperhomocysteinemia accompanying folate deficiency also imposes health risks on pregnant women. It might induce apoptosis and DNA damage in placental vascular cells and maternal endothelial malfunction leading to severe complications [89]. It was evident that hyperhomocysteinemia could increase the risk of pregnancy complications ca. 18-fold in the second trimester [90].

On the other hand, excess folic acid can lead to the T allele of methylene tetrahydrofolate in infants. Infants with this type of allele suffer from sudden abnormal neurological symptoms during adult life, including bipolar disorder, depression, and schizophrenia [86].

In neonates, infants, children, and adolescents, inborn folate transport and metabolism errors are often associated with several clinical manifestations. These include developmental delays, cognitive deterioration, motor and gait abnormalities, behavioral or psychiatric symptoms, seizures, signs of demyelination or failure of myelination, and vascular changes seen on magnetic resonance imaging or postmortem examination. Less commonly, subacute combined degeneration and peripheral neuropathy might also occur [91].

In addition, infants and children of women who suffered from folate deficiency during pregnancy were found to experience asthma in later stages of life [55]. However, the underlying mechanism is still unclear. In addition, folic acid deficiency might cause megaloblastic anemia and infant neural tube defects [87].

Adulthood and Elderly: Concerning folic acid, smoking changes folate storage and metabolism because the one-carbon metabolism is influenced by the redox balance, which is readily altered by smoking [89].

### 2.8. Cobalamin

Table 8 comprises the dietary sources, RDA, and main actions of vitamin B12.

Pregnancy: At the start of pregnancy, women must be supplemented with folic acid and vitamin B12 to meet the increased requirements of increased DNA, RNA, and protein synthesis. The recommended daily allowance of vitamin B12 is 2.6 mg/d for pregnant women and 2.8 mg/d for lactating women [92].

As previously mentioned, folate and cobalamin status of mothers during gestation highly affects the infant’s quality regarding both vitamins later in future life stages [85]. Additionally, it was observed that pregnant women with poor folate and cobalamin status suffer from high body mass indexes [92].

Low cobalamin levels and increased risk of low birth weight (LBW) are differentially related to the trimester during which the mother encountered vitamin B12 deficiency, i.e., the first-trimester lack can increase the risk of LBW up to 8 times [90]. The deficiency of vitamin B12 has also been related to other pregnancy complications such as recurrent miscarriages, preterm delivery, and intrauterine growth restriction [85].

Lactation: Breastfed infants’ cobalamin levels depended on their mothers’ status. In contrast, formula-fed infants were less at risk of deficiency as they were supplemented with all micronutrients meeting AI, which is 0.4–0.5 μg/day of cobalamin in the first year of life [93]. Breast milk vitamin B12 content varies according to maternal diet and cobalamin status; thus, nursing mothers following a vegan or macrobiotic diet should receive vitamin B12 supplements [92]. Vitamin B12 is limited in a vegan diet and should always be supplemented.

As with any other nutrient in breast milk, an infant experiences nutrient deficiency at six months, and breast milk nutrient content can no longer satisfy the baby’s nutritional daily requirements. Therefore, as weaning starts, vitamin B12-rich food such as meat and poultry should be included in the infant’s diet [92].

Infancy and childhood: Vitamin B12 deficiency in infants and children is manifested in the form of neurological, insufficient physical growth (failure to thrive), and hematological disorders, with most manifestations being found treatable except those affecting the nervous system, which might be irreversible. Such symptoms must be followed up closely, particularly in infants experiencing pathological malabsorption.

Some regimens involve the intramuscular injection of vitamin B12 (at a dose of 1 mg) and continue according to the response. The improvement of symptoms varies, with recovery from anemia found to be even faster with treatment with folic acid and iron supplements, including cobalamin [92].

In general, early childhood malnutrition has been related to poor cognitive function, school performance, and IQ scores in the short and long term. Deficiencies in various vitamins, such as vitamin B12, thiamine, and niacin, have been associated with cognitive impairment [94]. It should be noted that the recommended adequate intake of pantothenic acid is 1.7 to 5.0 mg/day for children, as it plays an essential role in normal development and growth [38]. Regarding vitamin B12, the child will continue to suffer from deficiency symptoms if it is not diagnosed early in infancy. As a result, school-aged children with cobalamin deficiency might suffer from altered motor development, cognitive disorders, and speech and language skills [92].

Adulthood: In addition, the clinical manifestation of cobalamin deficiency in adults resembles that in adulthood, which is highly heterogeneous, ranging from fatigue, common sensory neuropathy, neuropsychiatric symptoms, atrophic glossitis (Hunter’s glossitis), isolated macrocytosis and neutrophil hypersegmentation, to severe disorders, including combined sclerosis of the spinal cord, hemolytic anemia, and even pancytopenia [95].

Elderly: Vitamin B12 deficiency and/or age-related impairments in its function are increasingly recognized as contributing to age-related cognitive decline, subtle deficits, and frank dementia [96]. Regarding cobalamin, its deficiency results in disrupted cellular metabolism in all life cycles, in addition to age-related disease and functional decline, including cognition, cardiovascular disease, and bone health. Last but not least, it was postulated that a link exists between folic acid deficiency in older adults with homocysteine, aging, depression, dementia, and vascular disease [91].

## 3. Conclusions

Micronutrient intake is a crucial issue that should be considered. Despite the small amounts needed in DRI, they have inevitable metabolic functions and are involved in many enzymatic reactions as cofactors, maintaining health and preventing diseases. Thus, any imbalance, either deficiency or over-consumption, may cause a wide array of reversible and irreversible symptoms that may sometimes lead to death. Fortified foods can be a solution to prevent their deficiency. The RDA of micronutrients differs along the life cycle based on age, gender, ethnicity, physical activity level, etc., and RDAs generally increase by age to fulfill the growth and energy requirements. RDAs during pregnancy and lactation are even higher to sustain the milk’s increased demands and vitamin secretion. Thiamine, riboflavin, niacin, pyridoxine, and folic acid are crucial for maternal and fetus health. During infancy and childhood, B vitamins are integrated into physical and psychological development, which have a pivotal impact on one’s overall health in adolescence and adulthood. A higher intake of B vitamins in the elderly also prevents aging problems, especially inflammation-related. However, all supplementations should be carefully monitored to avoid overdoses and hypervitaminosis. Hence, more research should be invested to study each vitamin individually, concerning nutritional disparities in each life stage, with extensive attention paid to cultural differences and lifestyles.

## Figures and Tables

**Table 1:** Recommended daily intake (RDI), main action, and natural sources of vitamin B1 (thiamine) in different life stages.

Life Stage		RDI (mg/Day)	Importance and Health Effects	Deficiency Symptoms	Source
Infant	0–6 Months	0.2	Involved in carbohydrates and amino acids metabolism as well as neurotransmitters biosynthesis	Deficiency may cause irritability, vomiting, ataxia, altered sleep patterns, and may cause infant encephalopathy	Maternal Milk
	7–12 Months	0.2			
Children	1–3 Years	0.5		Deficiency may cause anorexia, irritability, agitation, muscle pain, diminished deep tendon reflexes, ataxia, paralysis, and pediatric depression	Meat, vegetables, whole grains, and legumes
	4–9 Years	0.9			
Teen	Girl	1.1			
	Boy	1.2			
Adult	Men	1.2		The deficiency is often associated with alcohol abuse causing Wernicke–Korsakoff syndrome	
	Women		Same as above Used to lessen the symptoms of premenstrual syndrome (PMS) and menorrhagia		
	Non-pregnant	1.0			
	Pregnant	1.3	Same as above	Deficiency cause *Hyperemesis gravidarum*	
	Lactating	1.5	Same as above Maternal supplements are recommended to avoid impaired infant growth		
Elderly		1.1–1.2	Same as above		

**Table 2:** Recommended daily intake (RDI), main action, and natural sources of vitamin B2 (riboflavin) in different life stages.

Life Stage		RDI (mg/Day)	Importance and Health Effects	Deficiency Symptoms	Source
Infant	0–6 Months	0.4	Early postnatal brain development Coenzyme in mitochondrial energy production in the flavoprotein redox reactions	Deficiency may cause abnormal brain development	Maternal Milk
	7–12 Months	0.3			
Children	1–3 Years	0.5	Same as above Treat migraines	Migraine, pain, and attention deficit hyperactivity disorder (ADHD)	Beef, organ meats—mostly in calf liver, egg, salmon, mushrooms, dark green leafy vegetables such as spinach.
	4–9 Years	0.9			
Teen	Girl	1.0			
	Boy	1.3			
Adult	Men	1.3	Same as above	Same as above	
	Women		Same as above Decrease incidence of PMS Cofactor in one-carbon metabolism		
	Non-pregnant	1.1		Risk of breast cancer	
	Pregnant	1.4	Same as above Reduce risk of preeclampsia Prevent mitochondrial dysfunction and oxidative stress Stabilize nitric oxide release	Preeclampsia	
	Lactating	1.6			
Elderly		1.1–1.3	Same as above Reduce the risk of Type 2 diabetes Antioxidant activity Reduce iron overload	Deficiency may elevate the risk of Type 2 diabetes	

**Table 3:** Recommended daily intake (RDI), main actions, and natural sources of vitamin B3 (niacin) in different life stages.

Life Stage		RDI (mg/Day)	Importance and Health Effects	Deficiency Symptoms	Source
Infant	0–6 Months	3	Play a major role in normal metabolic pathways Essential for normal development and growth Important for healthy cognitive function	Deficiency may cause Multiple congenital malformations	Maternal Milk
	7–12 Months	4-5			
Children	1–3 Years	6			Yeast, poultry, meat, Redfish (e.g., tuna and salmon), beans, and coffee.
	4–9 Years	8			
Teen	Girl	12			
	Boy	16			
Adult	Men	16	Same as above Prevent the occurrence of pellagra-associated dermatitis	Deficiency may cause pellagra-associated dermatitis	
	Women				
	Non-pregnant	14			
	Pregnant	18	Same as above Reduce the risk of multiple congenital malformations in the fetus		
	Lactating	17	Same as above	Same as above	
Elderly		14–16	Has neuroprotective effects against age-related disorders such as hearing loss and myelination	Deficiency may increase the incidence of hip fractures	

**Table 4:** Adequate daily intake (AI), main actions, and natural sources of vitamin B5 (pantothenic acid) in different life stages.

Life Stage		AI (mg/Day)	Importance and Health Effects	Deficiency Symptoms	Source
Infant	0–6 Months	1.7	Important for normal development and growth	Loss of appetite, growth impairment, dermatitis, weakness, ataxia, paralysis, adrenal hypertrophy, ulcers, and hepatic steatosis	Maternal Milk
	7–12 Months	3			
Children	1–3 Years	4			Organ meats (particularly liver and heart), broccoli, avocados, mushrooms, and some yeasts
	4–9 Years	4			
Teen	Girl	5			
	Boy	5			
Adult	Men	5	Inversely related to the levels of C-reactive protein (CRP)		
	Women				
	Non-pregnant	NA			
	Pregnant	5	Third-trimester pregnant women need to consume more than the average intake to maintain a blood vitamin level		
	Lactating	7	Same as above		
Elderly		5	Same as above		

NA: Not Available.

**Table 5:** Recommended daily intake (RDI), main actions, and natural sources of vitamin B6 (pyridoxine) in different life stages.

Life Stage		RDI (mg/Day)	Importance and Health Effects	Deficiency Symptoms	Source
Infant	0–6 Months	0.1	Essential for average infant growth in height and weight Lifelong treatment is essential for infants with an autosomal recessive defect in the glutamic acid decarboxylase enzyme	Deficiency may cause non-responsive polymorphic seizures	Maternal Milk
	7–12 Months	0.3			
Children	1–3 Years	0.5–0.6	Required for thymidine biosynthesis and host immunocompetence Treat the symptoms of behavioral disorders associated with autism, hyperkinetic syndrome, and schizophrenia. Adjuvant to anti-epileptic drugs Exert beneficial effects on stress accompanying adolescence phase		Cereals, fishes, meats, starchy vegetables such as potatoes, legumes; nuts, bananas, avocados, and non-citrus fruits
	4–9 Years	0.6			
Teen	Girl	1–1.2			
	Boy	1–1.3			
Adult	Men	1.3	Extremely beneficial against colorectal cancer in adult males Reduce plasma cholesterol level	microcytic hypochromic anemia Lymphopenia convulsions	
	Women				
	Non-pregnant	1.3	Necessary for estrogen metabolism Prescribed for women with breast cysts Effective during PMS		
	Pregnant	5.5–7.6	Pregnancy stabilization Prevent any miscarriages Improve hyperemesis gravidarum Essential for heme and porphyrin synthesis and the proper iron utilization by red blood cells Maintain normal fetal/infant development.	Hyperemesis gravidarum, anemia nausea, vomiting, spontaneous abortion	
	Lactating	5.5–7.6	Same as above Mood elevation Anemia improvement	Same as above	
Elderly		1.5–1.7	Reduce the risk of irritable bowel syndrome	Deficiency may cause irritable bowel syndrome	

**Table 6:** Adequate daily intake (AI), main actions, and natural sources of vitamin B7 (Biotin) in different life stages.

Life Stage		AI (µg/Day)	Importance and Health Effects	Deficiency Symptoms	Source
Infant	0–6 Months	5	Critical role in maintaining healthy hair, skin, and nails Prevent serious brain abnormalities	Insufficiency is linked to alopecia and dermatitis around body orifices	Maternal Milk
	7–12 Months	5–6			
Children	1–3 Years	8–12	Efficient in both shiny and opaque types of trachyonychia	Deficiency is linked to Trachyonychia	Red meat, eggs, nuts, seeds, and certain vegetables
	4–9 Years	12			
Teen	Girl	20–25			
	Boy	20–25			
Adult	Men	30	Treat the symptoms of inherited disorder biotinidase deficiency	Deficiency may cause myelopathy and irreversible neurological damages	
	Women				
	Non-pregnant	30			
	Pregnant	35			
	Lactating	35			
Elderly		30	It has a critical role in bone mineral homeostasis Has a role in allergic and autoimmune disorders	Deficiency is related to specific diseases such as diabetes mellitus, liver and skin disorders, immunological and neurological abnormalities, and epilepsy	

**Table 7:** Recommended daily intake (RDI), main actions, and natural sources of vitamin B9 (folic acid) in different life stages.

Life Stage		RDI (mg/Day)	Importance and Health Effects	Deficiency Symptoms	Source
Infant	0–6 Months	0.05	Important for the proper development Protect from asthma in later stages of life.	Deficiency may cause long-term impairment of cognitive function, learning, and memory deficits and detected brain atrophy	Maternal Milk
	7–12 Months	0.08			
Children	1–3 Years	0.15	Essential for normal cognitive, motor, behavioral and vascular development	Deficiency may cause developmental delay, cognitive deterioration, motor and gait abnormalities, behavioral or psychiatric symptoms, seizures, signs of demyelination or failure of myelination, vascular changes, megaloblastic anemia and infant neural tube defects	Beans, lentils, leafy green vegetables, and lemons
	4–9 Years	0.2			
Teen	Girl	0.4			
	Boy	0.4			
Adult	Men	0.4	Modify the adverse effects of smoking regarding one-carbon metabolism and redox balance.		
	Women				
	Non-pregnant	0.4			
	Pregnant	0.6	It has a significant role in the normal neural and physical development of the fetus Prevent megaloblastic anemia and neural tube defects	In mothers, a deficiency might cause metabolic effects such as insulin resistance, glomerular sclerosis, neuropathy in the extremities, and megaloblastic anemia. Also, severe fetal adverse effects, including congenital neural tube defects, cardiac and urinary tract defects, and even cancer	
	Lactating	0.5	Same as above		
Elderly		0.4	Reduces the risk of depression, dementia, and vascular disease.		

**Table 8:** Adequate daily intake (AI), main actions, and natural sources of vitamin B12 (cobalamin) in different life stages.

Life Stage		AI (µg/Day)	Importance and Health Effects	Deficiency Symptoms	Source
Infant	0–6 Months	0.4	Maintain normal physical growth Prevent neurological and hematological disorders	Deficiency may cause neurological, insufficient physical growth (failure to thrive), and hematological disorders	Maternal Milk
	7–12 Months	0.5			
Children	1–3 Years	0.7	Essential for healthy cognitive function, motor development, speech and language skills		Fish, meat, dairy products such as cheese and eggs
	4–9 Years	1.2			
Teen	Girl	1.8–2.4			
	Boy	1.8–2.4			
Adult	Men	2.4	Reduces the deficiency manifestation ranging from fatigue to severe disorders	Deficiency may cause fatigue, common sensory neuropathy, neuropsychiatric symptoms, atrophic glossitis, isolated macrocytosis, neutrophil hypersegmentation, combined sclerosis of the spinal cord, hemolytic anemia, and pancytopenia	
	Women				
	Non-pregnant	2.4			
	Pregnant	2.6	Cobalamin status of mothers during gestation highly affects the infant’s status	Deficiency may cause high body mass indexes, recurrent miscarriages, preterm delivery, and intrauterine growth restriction	
	Lactating	2.8	Same as above	Same as above	
Elderly		2.4	Reduces the risk of disrupted cellular metabolism, age-related disease, and functional decline, including cognition, cardiovascular disease, and bone health.	Deficiency may contribute to age-related cognitive decline, subtle deficits, frank dementia, cardiovascular disease, and bone health.	

## Data Availability

Data can be demanded upon request.

## Abbreviations

5HT	Serotonin
Ach	Acetylcholine
AD	Alzheimer’s disease
AI	Adequate intake
ALP	Alkaline phosphatase
CoA	Coenzyme A
DFE	Dietary Folate Equivalents
FAD	Flavin adenine dinucleotide
FMN	Flavin mononucleotide
Hcy	Homocysteine
HG	Hyperemesis gravidarum
HLCS	holocarboxylase synthetase (HLCS)
KS	Korsakoff syndrome
LBW	Low birth weight
Mg	Magnesium
NAD	Nicotinamide adenine dinucleotide
NADP	Nicotinamide adenine dinucleotide phosphate
NAMPT	Nicotinamide phosphoribosyltransferase
NE	Niacin equivalents
NTG	normal-tension glaucoma
OCAs	Oral contraceptive agents
PDH	Pyruvate dehydrogenase
PL	Pyridoxal
PM	Pyridoxamine
PMS	Premenstrual syndrome
PN	Pyridoxine
PNG	Pyridoxine-59-b-D-glucoside
RBC	Red blood cell
RDA	Recommended dietary allowance
RDI	Recommended dietary intake
SIDS	Sudden infant death syndrome
TDP	Thiamine diphosphate
TPP	Thiamine pyrophosphate
WE	Wernicke’s encephalopathy
α-KGDH	α-ketoglutarate dehydrogenase
